# Supplementary material for: Serological responses and vaccine effectiveness for extended COVID-19 vaccine schedules in England
Source: Nat Commun. 2021 Dec 10;12:7217. doi: 10.1038/s41467-021-27410-5 (PMC8664823; doi:10.1038/s41467-021-27410-5)
Supplement: Supplementary file 1 — Supplementary Information [file 41467_2021_27410_MOESM1_ESM.pdf]

Supplementary Material

Supplementary Table 1: Demographics of individuals in the CONSENSUS study, by vaccine and age group.

Vaccine & age group	contribution	N	age	sex (if known)	ethnicity (if known)	past infection
			median (IQR)	n male (%)	n non-white (%)	n (%)
AstraZeneca, extended schedule, ages 50-64	all recruited	170	54 (52 - 57)	81 (48.8%)	89 (52.4%)	56 (32.9%)
	≥2 dose 1 samples	111	55 (53 - 58)	51 (46.4%)	48 (43.2%)	36 (32.4%)
	≥1 dose 2 sample	80	54 (53 - 57.5)	39 (49.4%)	26 (32.5%)	23 (28.8%)
AstraZeneca, extended schedule, ages 65-79	all recruited	147	70 (68 - 73)	68 (46.3%)	36 (24.7%)	25 (17%)
	≥2 dose 1 samples	121	70 (68 - 73)	54 (44.6%)	26 (21.7%)	20 (16.5%)
	≥1 dose 2 sample	111	70 (68 - 73)	47 (42.3%)	19 (17.3%)	16 (14.4%)
Astra Zeneca, extended schedule, ages 80-89	all recruited	12	84 (82 - 85.5)	5 (41.7%)	2 (16.7%)	1 (8.3%)
	≥2 dose 1 samples	10	83 (82 - 85)	3 (30%)	1 (10%)	1 (10%)
	≥1 dose 2 sample	9	82 (82 - 84)	3 (33.3%)	1 (11.1%)	1 (11.1%)
Pfizer, extended schedule, ages 50-64	all recruited	1	61	0		0
	≥2 dose 1 samples	1	61	0		0
	≥1 dose 2 sample	1	61	0		0
Pfizer, extended schedule, ages 65-79	all recruited	260	72 (69 - 74)	117 (45%)	28 (10.9%)	26 (10%)
	≥2 dose 1 samples	211	71 (69 - 74)	95 (45%)	19 (9.1%)	19 (9%)
	≥1 dose 2 sample	225	72 (69 - 74)	102 (45.3%)	22 (9.9%)	21 (9.3%)
Pfizer, extended schedule, ages 80-89	all recruited	72	82 (81 - 84)	31 (43.1%)	7 (9.9%)	9 (12.5%)
	≥2 dose 1 samples	63	82 (81 - 84)	25 (39.7%)	6 (9.7%)	7 (11.1%)
	≥1 dose 2 sample	56	83 (81 - 84)	22 (39.3%)	4 (7.3%)	5 (8.9%)
Pfizer, standard schedule, ages 65-79	all recruited	59	75 (74 - 77)	28 (47.5%)	6 (10.3%)	7 (11.9%)
	≥2 dose 1 samples	1	66	0	0	0
	≥1 dose 2 sample	59	75 (74 - 77)	28 (47.5%)	6 (10.3%)	7 (11.9%)
Pfizer, standard schedule, ages 80-89	all recruited	29	82 (81 - 85)	14 (48.3%)	3 (10.3%)	1 (3.4%)
	≥2 dose 1 samples	0				
	≥1 dose 2 sample	28	82 (80.5 - 85)	14 (50%)	3 (10.7%)	0 (0%)

Supplementary Table 2: Test negative case control results showing adjusted odds ratios (OR) and vaccine effectiveness (VE) post first dose and post second dose according to intervals between doses for Pfizer vaccine in different age cohorts.

	Pfizer dose	days between doses	days since dose	controls	cases	OR (95% CI)	VE (95% CI)
Age 80+ and first vaccine dose before January 4th 2021 or unvaccinated. Cases and controls from Dec 8th 2020			unvaccinated	15936	9178	base	base
	1		0-3	281	172	1.23 (1.01-1.5)	
			4-13	862	731	1.29 (1.16-1.44)	
			14-27	754	467	0.9 (0.8-1.02)	10% (-2-20)
			>=28	1528	269	0.45 (0.38-0.53)	55% (47-62)
	2	19-29	>=14	2219	144	0.21 (0.17-0.26)	79% (74-83)
		30-44	>=14	6	0		
		45-64	>=14	22	0		
		65-84	>=14	522	4	0.1 (0.03-0.28)	90% (72-97)
		85+	>=14	13	0		
Age 80+ and first vaccinated from Jan 4th 2021 or unvaccinated, cases and controls from Jan 4th 2021			unvaccinated	5676	3881	base	base
	1		0-3	424	267	0.98 (0.83-1.15)	
			4-13	1277	699	0.93 (0.83-1.05)	
			14-27	1580	398	0.59 (0.51-0.68)	41% (32-49)
			>=28	3315	385	0.58 (0.48-0.69)	42% (31-52)
	2	19-29	>=14	212	22	0.23 (0.12-0.44)	77% (56-88)
		30-44	>=14	5	0		
		45-64	>=14	42	1	0.18 (0.02-1.34)	82% (-34-98)
		65-84	>=14	1289	30	0.1 (0.06-0.17)	90% (83-94)
		85+	>=14	83	2	0.06 (0.01-0.27)	94% (73-99)
Age 65-79 and first vaccinated from Jan 4th 2021 or unvaccinated, cases and controls from Jan 4th 2021			unvaccinated	76821	26463	base	base
	1		0-3	1536	600	1.3 (1.18-1.43)	
			4-13	4338	1242	1.06 (0.99-1.14)	
			14-27	4896	640	0.57 (0.52-0.63)	43% (37-48)
			>=28	11053	745	0.47 (0.42-0.52)	53% (48-58)
	2	19-29	>=14	388	33	0.23 (0.15-0.34)	77% (66-85)
		30-44	>=14	49	0		
		45-64	>=14	483	7	0.08 (0.04-0.17)	92% (83-96)
		65-84	>=14	5247	118	0.11 (0.08-0.14)	89% (86-92)
		85+	>=14	174	7	0.14 (0.06-0.3)	86% (70-94)
Age 50-64 and first vaccinated from Feb 1st 2021 or unvaccinated, cases and controls from Feb 1st 2021			unvaccinated	136905	33904	base	base
	1		0-3	1478	377	1.09 (0.97-1.22)	
			4-13	3599	831	1.04 (0.96-1.12)	
			14-27	4116	457	0.53 (0.48-0.59)	47% (41-52)
			>=28	9300	702	0.49 (0.45-0.53)	51% (47-55)
	2	19-29	>=14	83	4	0.12 (0.04-0.33)	88% (67-96)
		30-44	>=14	114	7	0.26 (0.12-0.56)	74% (44-88)
		45-64	>=14	643	24	0.11 (0.08-0.17)	89% (83-92)
		65-84	>=14	3977	127	0.08 (0.06-0.09)	92% (91-94)
		85+	>=14	110	3	0.06 (0.02-0.19)	94% (81-98)

Supplementary Table 3: Test negative case control results showing adjusted odds ratios (OR) and vaccine effectiveness (VE) post first dose and post second dose according to intervals between doses for Astra Zeneca vaccine in different age cohorts.

	AZ dose	days between doses	days since dose	controls	cases	OR (95% CI)	VE (95% CI)
Age 80+ and first vaccinated from Jan 4th 2021 or unvaccinated , cases and controls from Jan 4th 2021			unvaccinated	5676	3881	base	base
	1		0-3	424	228	0.74 (0.62-0.88)	
			4-13	853	545	1.03 (0.9-1.19)	
			14-27	957	333	0.78 (0.66-0.93)	22% (7-34)
			>=28	1986	237	0.58 (0.47-0.71)	42% (29-53)
	2	19-29	>=14	53	1	0.04 (0-0.28)	96% (72-100)
		30-44	>=14	5	1		
		45-64	>=14	92	1	0.04 (0.01-0.32)	96% (68-99)
		65-84	>=14	574	31	0.18 (0.11-0.32)	82% (68-89)
		85+	>=14	39	2	0.12 (0.03-0.52)	88% (48-97)
Age 65-79 and first vaccinated from Jan 4th 2021 or unvaccinated , cases and controls from Jan 4th 2021			unvaccinated	76821	26463	base	base
	1		0-3	3692	636	0.68 (0.62-0.75)	
			4-13	6261	1631	1.1 (1.03-1.17)	
			14-27	7070	937	0.67 (0.61-0.73)	33% (27-39)
			>=28	17000	1045	0.48 (0.44-0.54)	52% (46-56)
	2	19-29	>=14	247	30	0.34 (0.23-0.53)	66% (47-77)
		30-44	>=14	101	4	0.27 (0.1-0.75)	73% (25-90)
		45-64	>=14	945	68	0.39 (0.3-0.53)	61% (47-70)
		65-84	>=14	7556	435	0.26 (0.21-0.31)	74% (69-79)
		85+	>=14	270	20	0.27 (0.17-0.44)	73% (56-83)
Age 50-64 and first vaccinated from Feb 1st 2021 or unvaccinated , cases and controls from Feb 1st 2021			unvaccinated	136905	33904	base	base
	1		0-3	6895	1012	0.7 (0.65-0.75)	
			4-13	12120	2584	1.1 (1.05-1.16)	
			14-27	14597	1491	0.62 (0.58-0.66)	38% (34-42)
			>=28	34222	3633	0.58 (0.54-0.61)	42% (39-46)
	2	19-29	>=14	84	18	0.48 (0.29-0.82)	52% (18-71)
		30-44	>=14	219	33	0.45 (0.31-0.66)	55% (34-69)
		45-64	>=14	2311	313	0.3 (0.26-0.34)	70% (66-74)
		65-84	>=14	7443	782	0.23 (0.21-0.26)	77% (74-79)
		85+	>=14	165	25	0.3 (0.19-0.46)	70% (54-81)
