# Supplementary material for: Serological responses and vaccine effectiveness for extended COVID-19 vaccine schedules in England
Source: Nat Commun. 2021 Dec 10;12:7217. doi: 10.1038/s41467-021-27410-5 (PMC8664823; doi:10.1038/s41467-021-27410-5)
Supplement: Supplementary file 3 — Reporting Summary [file 41467_2021_27410_MOESM3_ESM.pdf]

nature portfolio

Corresponding author(s): NMED-A114969

Last updated by author(s): 2021/08/02

Reporting Summary

Nature Portfolio wishes to improve the reproducibility of the work that we publish. This form provides structure for consistency and transparency in reporting. For further information on Nature Portfolio policies, see our Editorial Policies and the Editorial Policy Checklist.

Statistics

For all statistical analyses, confirm that the following items are present in the figure legend, table legend, main text, or Methods section.

n/a	Confirmed	
☐	☒	The exact sample size (*n*) for each experimental group/condition, given as a discrete number and unit of measurement
☐	☒	A statement on whether measurements were taken from distinct samples or whether the same sample was measured repeatedly
☐	☒	The statistical test(s) used AND whether they are one- or two-sided<br>*Only common tests should be described solely by name; describe more complex techniques in the Methods section.*
☐	☒	A description of all covariates tested
☒	☐	A description of any assumptions or corrections, such as tests of normality and adjustment for multiple comparisons
☐	☒	A full description of the statistical parameters including central tendency (e.g. means) or other basic estimates (e.g. regression coefficient) AND variation (e.g. standard deviation) or associated estimates of uncertainty (e.g. confidence intervals)
☐	☒	For null hypothesis testing, the test statistic (e.g. F, t, r) with confidence intervals, effect sizes, degrees of freedom and P value noted<br>*Give P values as exact values whenever suitable.*
☒	☐	For Bayesian analysis, information on the choice of priors and Markov chain Monte Carlo settings
☒	☐	For hierarchical and complex designs, identification of the appropriate level for tests and full reporting of outcomes
☐	☒	Estimates of effect sizes (e.g. Cohen's *d*, Pearson's *r*), indicating how they were calculated

Our web collection on statistics for biologists contains articles on many of the points above.

Software and code

Policy information about availability of computer code

Data collection	no software was used
Data analysis	STATA version 14.2 (STATAcorp, Texas)

For manuscripts utilizing custom algorithms or software that are central to the research but not yet described in published literature, software must be made available to editors and reviewers. We strongly encourage code deposition in a community repository (e.g. GitHub). See the Nature Portfolio guidelines for submitting code & software for further information.

Data

Policy information about availability of data

All manuscripts must include a data availability statement. This statement should provide the following information, where applicable:

- Accession codes, unique identifiers, or web links for publicly available datasets
- A description of any restrictions on data availability
- For clinical datasets or third party data, please ensure that the statement adheres to our policy

Applications for relevant anonymised data should be submitted to the Public Health England Office for Data Release.

Field-specific reporting

Life sciences study design

All studies must disclose on these points even when the disclosure is negative.

Sample size	Sample size was determined based on providing reasonable precision for estimates of geometric mean concentrations at each time point, age group, interval between doses and vaccine type for those not previously infected. To calculate precision an estimate of the standard deviation of responses post vaccination was required. In the absence of data post vaccination this was estimated to be 0.5 log10 units based on data on responses post natural infection. Using this estimate, and considering that recruitment may be variable in different groups the 95% confidence interval +/- fold-widths were estimated to be +/- 54%, +/- 39%, and +/-26% for sample sizes of 30,50 and 100. For vaccine effectiveness sample size was based on all available pillar 2 symptomatic cases and controls.
Data exclusions	persons <50 years, unable to provide written consent, had taken part in COVID-19 vaccine trials or were clinically vulnerable were excluded from participating in the CONSENSUS study. For vaccine effectiveness cases and controls who were not symptomatic or who had previously tested positive were excluded.
Replication	Samples were tested as singletons only on validated SARS-CoV-2 antibody assays.
Randomization	Consensus: Participants were not blinded/randomised, as it was an audit of the current vaccine schedule and we were not able to make clinical decisions on which vaccine participants received. VE: randomisation/blinding not possible as used surveillance data. Adjustment for covariates as documented in the manuscript was done in multivariable analyses.
Blinding	Consensus: Participants were not blinded/randomised, as it was an audit of the current vaccine schedule and we were not able to make clinical decisions on which vaccine participants received. VE: randomisation/blinding not possible as used surveillance data.

Reporting for specific materials, systems and methods

We require information from authors about some types of materials, experimental systems and methods used in many studies. Here, indicate whether each material, system or method listed is relevant to your study. If you are not sure if a list item applies to your research, read the appropriate section before selecting a response.

Materials & experimental systems

n/a	Involved in the study	
☒	☐	Antibodies
☒	☐	Eukaryotic cell lines
☒	☐	Palaeontology and archaeology
☒	☐	Animals and other organisms
☐	☒	Human research participants
☒	☐	Clinical data
☒	☐	Dual use research of concern

Methods

n/a	Involved in the study	
☒	☐	ChIP-seq
☒	☐	Flow cytometry
☒	☐	MRI-based neuroimaging

Human research participants

Policy information about studies involving human research participants

Population characteristics	We recruited 750 participants aged 50-89 years (median age, 71, IQR 66-76 years)- 421 received at least one BNT162b2 dose and 329 at least one AZD1222 dose (Table 1). Overall, 46% (344/746) were male, 27% (171/743) were of non-White ethnicity, 16.8% (126/750) had evidence of previous infection at enrollment and one seroconverted during the study. Adults aged 50-64 years were more likely to have evidence of previous infection than older adults (56/171; 32.8% vs 70/579; 12.1%; p<0.001).
Recruitment	Potential participants will be recruited through NHS hospitals and General Practices in England. All participants will have the opportunity to ask questions about the audit and provide written informed consent before taking part.
Ethics oversight	The CONSENSUS study/audit was approved by PHE's R&D Research Ethics and Governance Group. No: NR0253

Note that full information on the approval of the study protocol must also be provided in the manuscript.
